# Cardiac sarcoidosis: from clinical manifestations to heart transplantation

**DOI:** 10.3389/fmed.2026.1723644

**Published:** 2026-03-10

**Authors:** Anna Starshinova, Petr Fedotov, Musaeva Bulgun, Igor Kudryavtsev, Artem Rubinstein, Arthur D. Aquino, Dmitry Kudlay, Evgeny Shlyakhto

**Affiliations:** 1 Almazov National Medical Research Centre, Saint Petersburg, Russia; 2 Laboratory of Probabilistic Methods in Analysis, Faculty of Mathematics and Computer Science, Saint Petersburg State University, Saint Petersburg, Russia; 3 Institute of Experimental Medicine, Saint Petersburg, Russia; 4 Department of Pharmacology, Institute of Pharmacy, I.M. Sechenov First Moscow State Medical University, Moscow, Russia; 5 Institute of Immunology FMBA of Russia, Moscow, Russia; 6 Department of Pharmacognosy and Industrial Pharmacy, Faculty of Fundamental Medicine, Lomonosov Moscow State University, Moscow, Russia

**Keywords:** cardiac sarcoidosis, granulomatous myocarditis, heart transplantation, inflammasome, Th17.1

## Abstract

**Background:**

Cardiac sarcoidosis (CS) represents one of the most severe and prognostically unfavorable manifestations of systemic sarcoidosis. Its diagnosis is often delayed due to non-specific symptoms and the patchy myocardial distribution of granulomatous inflammation.

**Objectives:**

To summarize the current understanding of epidemiology, diagnostic strategies, immunopathology, and therapeutic advances in CS, and to propose recommendations for future research and clinical management.

**Methods/scope:**

We analyze epidemiological data, autopsy series, and clinical cohorts to estimate the true prevalence and spectrum of CS. We review diagnostic algorithms combining electrocardiographic, echocardiographic, cardiac MRI, and 18F-FDG PET imaging with histopathological methods. Immunopathological mechanisms are discussed, with particular focus on Th17.1 cells, M2 macrophage polarization, and inflammasome activation. Therapeutic modalities – including corticosteroids, immunosuppressants, biologics (e.g., TNF inhibitors, IL-1/IL-18 blockers), and mechanical support (LVAD, transplantation) – are critically appraised based on existing clinical and registry evidence.

**Results:**

Morphological evidence suggests cardiac involvement in 20%–30% of sarcoidosis cases, yet clinically manifest CS is diagnosed in only ∼5%. Advanced imaging has increased detection of subclinical disease. Th17.1 cells and M2 macrophages appear central in granuloma formation and fibrotic progression, while activation of the NLRP3 inflammasome represents a promising therapeutic target. Corticosteroids remain the first-line therapy; steroid-sparing immunosuppression and biological therapies are under investigation. Heart transplantation yields favorable long-term outcomes in CS, with low rates of rejection and recurrence when accompanied by appropriate surveillance.

**Conclusion:**

A multifaceted diagnostic and therapeutic approach is essential for CS. Prospective trials are urgently needed to validate biomarkers, optimize immunomodulatory regimens, and test targeted interventions (e.g., IL-1/IL-18 blockade, NLRP3 inhibition). In advanced disease, transplantation remains a viable and effective option. Concerted efforts in mechanistic research, biomarker discovery and multicenter clinical trials will be critical to improving prognosis in cardiac sarcoidosis.

## 1 Introduction

Sarcoidosis remains an etiologically unclear disease, with a key pathomorphological hallmark–the non-caseating granuloma–arising under conditions of immune dysregulation. The epidemiology of the disease varies across regions and ethnic groups, ranging from several dozen cases per 100,000 in Northern Europe and some regions of Russia to only a few cases in Asian countries. The peak incidence occurs among young and middle-aged adults (25–40 years), with a second peak observed in women after the age of 50. The disease is more common in women, and chronic forms with extrapulmonary involvement have a sustained negative impact on quality of life, particularly in individuals exposed to occupational hazards (1, 2). Sarcoidosis is characterized by a high variability in the number of organs involved in the pathological process, which considerably increases diagnostic uncertainty. The clinical course of the disease may be acute, subacute, or chronic, and its outcomes range from spontaneous remission to a disabling chronic course. The most severe complications include pulmonary fibrosis, renal failure, cardiac involvement, neurosarcoidosis, lupus pernio, and vision loss resulting from posterior uveitis (3).

As the clinical manifestations of sarcoidosis are often non-specific, histological examination of granulomas is required in most cases to confirm the diagnosis.

It has been hypothesized that sarcoidosis represents an autoimmune disorder, in which the key pathogenic mechanism involves the interaction of an exogenous or endogenous antigen with components of the innate immune system, primarily macrophages and dendritic cells (4). Chronic sarcoidosis develops in approximately 10%–30% of patients and is characterized by the persistence of clinical manifestations and morphological signs of granulomatous inflammation for more than 2–5 years It is assumed that the granulomatous process is triggered by contact with one or several antigens possessing immunostimulatory potential. Possible triggers include infectious agents, vaccine components, and various inorganic substances (5).

The disease exhibits variable clinical courses that may include:

- Progressive pulmonary involvement with the development of fibrosis and respiratory failure;
- Cardiac complications (arrhythmias, heart failure, risk of sudden death);
- Neurological manifestations (neurosarcoidosis);
- Ophthalmological disorders (chronic uveitis with risk of vision loss);
- Systemic complications, including renal and cutaneous involvement.
- The major complications of chronic sarcoidosis include (6, 7):
- Pulmonary fibrosis (up to 25% of cases);
- Severe cardiac manifestations (5%–7%);
- Renal failure (3%–5%);
- Vision loss due to uveitis.

The prognosis of chronic sarcoidosis largely depends on the localization of organ involvement: cardiac, neurological, and pulmonary forms are associated with the highest risks of disability and mortality. Chronic sarcoidosis is defined as a disease characterized by persistent inflammation and the formation of specific granulomas capable of affecting multiple organs and systems. Despite extensive research, many pathogenic mechanisms and clinical patterns remain insufficiently understood (1, 4).

Clinical manifestations of chronic sarcoidosis vary according to the localization of the lesions and individual patient characteristics. Although the most typical combinations of organ involvement have been described, they do not allow for reliable prediction of disease progression. Nevertheless, their identification underscores the need for thorough and comprehensive evaluation, both in newly diagnosed sarcoidosis and in long-standing involvement of a single organ.

The diagnosis of chronic sarcoidosis remains a complex task, requiring differential investigation to exclude other possible causes of pathological changes.

Among the complications of chronic sarcoidosis, cardiac involvement occupies a special place. It may remain subclinical but often leads to life-threatening conditions, including severe arrhythmias, progressive heart failure, and sudden cardiac death. For this reason, cardiac sarcoidosis is regarded as one of the most severe and prognostically unfavorable manifestations of systemic disease, demanding particular attention in both clinical diagnosis and the selection of optimal therapeutic strategies. Sarcoidosis of the heart is a significant and potentially fatal manifestation of systemic sarcoidosis, characterized by the formation of non-caseating granulomas that progress and cause inflammation across all three layers of the heart, most commonly affecting the myocardium.

## 2 Identification of cardiac sarcoidosis

Over the past 25 years, the detection of cardiac sarcoidosis (CS) has increased significantly, primarily due to advances in diagnostic methods, including modern imaging techniques (8). Approximately 5% of patients with sarcoidosis present with clinically evident cardiac involvement. In such cases, cardiac manifestations often predominate over extracardiac ones; however, in up to 20% of patients, the disease remains asymptomatic (9).

The prevalence of cardiac sarcoidosis varies substantially depending on the population, geographical region, and diagnostic criteria applied. According to autopsy data, morphological evidence of cardiac involvement is found in 20%–30% of patients with systemic sarcoidosis, whereas clinically significant manifestations during life are observed in only about 5% (10). With the introduction of highly sensitive imaging modalities, such as contrast-enhanced cardiac magnetic resonance imaging (MRI) and 18F-fluorodeoxyglucose positron emission tomography (FDG-PET), the frequency of detected cardiac involvement has markedly increased, particularly in subclinical cases (11, 12).

Epidemiological studies indicate considerable geographic variation: in Japan, the frequency of cardiac involvement in sarcoidosis reaches 25%–58% (5, 13), whereas in Europe and North America it is 5%–10% (14). The overall prevalence of CS in the general population is estimated at 2–7 cases per 100,000 people (15); however, the true incidence is likely higher due to the large number of undiagnosed subclinical forms (Table 1).

**TABLE 1:** Global detection rates of cardiac sarcoidosis.

Diagnostic method	Prevalence of cardiac sarcoidosis
Clinical manifestations	≈ 5%–7% of patients with sarcoidosis
Subclinical forms (screening with PET-CT/MRI)	≈ 25%–30%
Autopsy data (USA)	Up to 20%–29%
Autopsy data (Japan)	Up to 58%–70%

It is estimated that up to 50% of cardiac sarcoidosis cases remain unrecognized during life (16).

### 2.1 Clinical manifestations

Sarcoidosis is highly heterogeneous in terms of anatomical localization, rate of disease progression, and response to immunomodulatory therapy, which complicates accurate diagnosis and clinical monitoring.

Cardiac manifestations depend on the localization and activity of the disease. They may range from an asymptomatic course to ventricular tachycardia, atrioventricular (AV) block, heart failure, or sudden cardiac death. Supraventricular arrhythmias are less frequent.

The diagnosis of cardiac sarcoidosis presents considerable challenges due to its phenotypic and histological overlap with other inflammatory cardiac diseases. The diagnosis should be considered in patients with extracardiac sarcoidosis, irrespective of the presence of cardiac symptoms. Two categories of diagnostic criteria are required: histological and clinical.

The histological criterion involves the detection of non-caseating epithelioid granulomas in an endomyocardial biopsy or other surgical specimen (1).

### 2.2 Proposed diagnostic algorithm for cardiac sarcoidosis

Step 1. Clinical Suspicion

Cardiac sarcoidosis should be suspected in the following clinical scenarios:

- Established extracardiac sarcoidosis, irrespective of cardiac symptoms
- Unexplained high-grade AV block (especially in patients < 60 years)
- Sustained or recurrent ventricular tachycardia of unclear etiology
- Unexplained heart failure (HFrEF or HFpEF)
- Syncope or aborted sudden cardiac death without alternative explanation

Step 2. Initial Cardiac Assessment

All patients with suspected cardiac involvement should undergo:

- 12-lead ECG
- Transthoracic echocardiography
- Ambulatory ECG (Holter) monitoring (24–72 h or extended monitoring)

Key findings prompting further evaluation include:

- AV conduction abnormalities
- Ventricular arrhythmias or frequent premature ventricular complexes
- Regional wall motion abnormalities
- Reduced or borderline left ventricular ejection fraction

Step 3. Advanced Cardiac Imaging

If initial evaluation is abnormal or clinical suspicion remains high:

Cardiac Magnetic Resonance (CMR)

- Assessment of myocardial structure and function
- Detection of late gadolinium enhancement (LGE)
- Typical patterns: mid-myocardial or subepicardial LGE, often involving basal septum or lateral wall

LGE presence is associated with:

- Increased risk of ventricular arrhythmias
- Adverse prognosis, even with preserved LVEF

FDG-PET/CT

- Evaluation of active myocardial inflammation
- Useful for:
  - distinguishing active inflammation from fibrosis
  - guiding immunosuppressive therapy
  - monitoring treatment response

Step 4. Diagnostic Classification

Diagnosis of cardiac sarcoidosis is established based on:

- Histological confirmation
  - Non-caseating granulomas on endomyocardial biopsy or extracardiac tissue
- Clinical diagnosis
  - Typical cardiac manifestations + extracardiac sarcoidosis
  - Supportive findings on CMR and/or FDG-PET

Given the low sensitivity of endomyocardial biopsy, a clinical diagnosis supported by multimodal imaging is often sufficient.

Step 5. Phenotype-Based Risk Stratification

Patients should be classified according to dominant clinical phenotype:

Arrhythmic phenotype

- VT/VF
- High-grade AV block
- Extensive LGE or RV involvement

→ Consider ICD, EPS, close rhythm surveillance

Heart failure phenotype

- Reduced or preserved LVEF
- Ventricular remodeling

→ Optimized HF therapy + immunosuppression if active inflammation

Inflammatory-active phenotype

- FDG uptake without extensive fibrosis

→ Immunosuppressive therapy escalation

Step 6. Follow-Up and Monitoring

- Serial ECG and Holter monitoring
- Repeat CMR and/or FDG-PET to assess disease activity
- Reassessment of arrhythmic and heart failure risk over time

**FIGURE 1:**
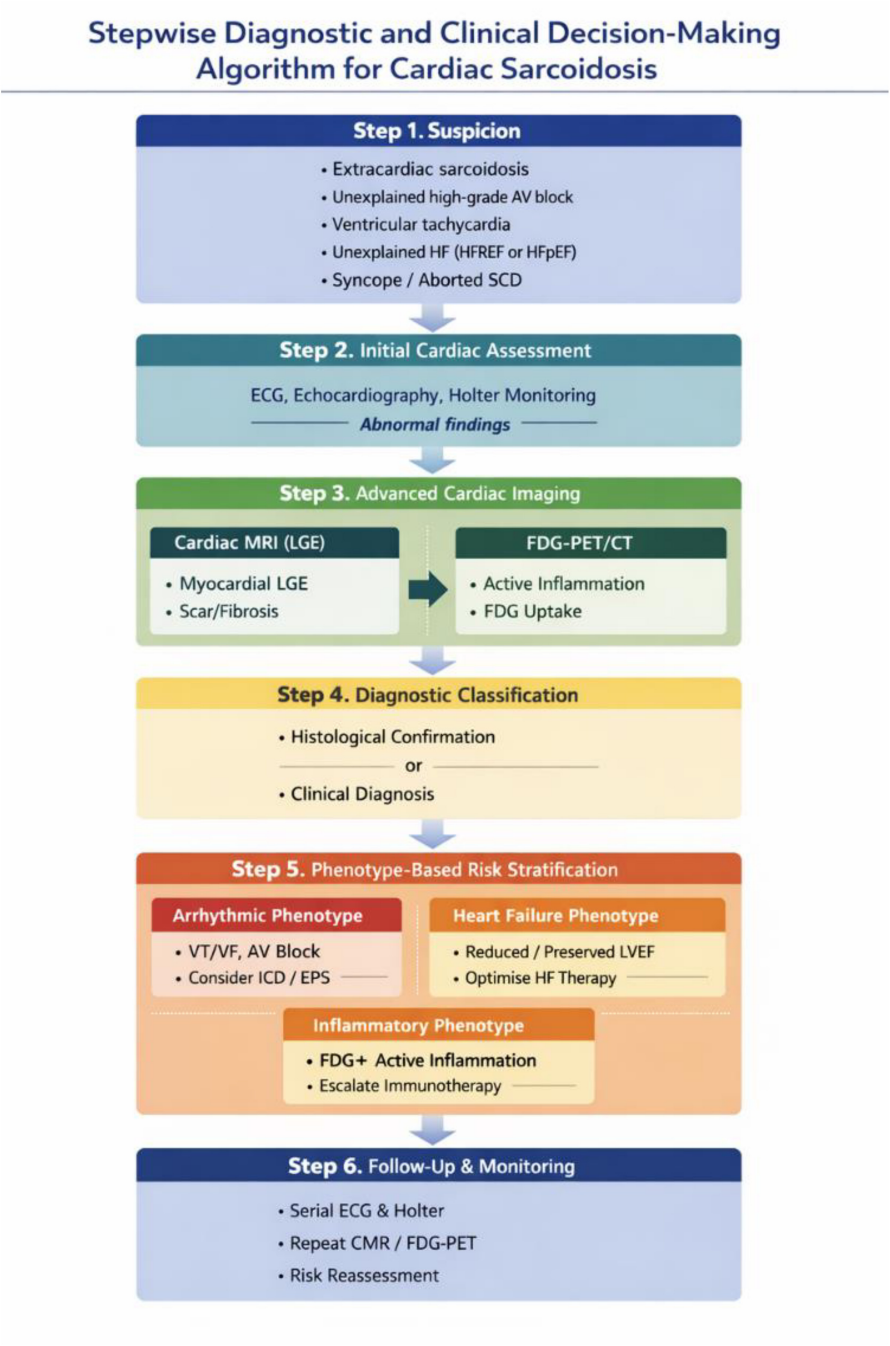
Stepwise diagnostic and clinical decision-making algorithm for cardiac sarcoidosis.

Clinical and functional manifestations of cardiac sarcoidosis are highly variable, ranging from mild symptoms to severe, life-threatening conditions, including sudden cardiac death (Figure 1).

Certain clinical syndromes necessitate the exclusion of cardiac sarcoidosis (Table 2).

**TABLE 2:** Examples of clinical syndromes requiring exclusion of cardiac sarcoidosis.

Syndrome	Typical features
Isolated AV block	Often in young patients without structural abnormalities; CS should be ruled out
Ventricular tachycardia	Mono- or polymorphic; treatment-resistant; risk of SCD
Unexplained heart failure	Reduced ejection fraction in the absence of ischemia or hypertension
Sudden death syndrome	History of syncope, ventricular fibrillation, or sustained VT

Electrocardiographic abnormalities are typically present in patients with clinically manifest cardiac sarcoidosis. These may include conduction defects of varying degrees, isolated bundle branch block, pathological Q waves (pseudo-infarct pattern), ST-T wave abnormalities, and, rarely, epsilon waves. Among asymptomatic patients, such abnormalities are found in only 3.2%–8.6% (10).

Clinical findings such as palpitations, syncope, atypical chest pain, dyspnea, reduced exercise tolerance, and physical signs (jugular venous distension, cardiac murmurs, gallop rhythm, or displaced apical impulse), as well as unexplained heart failure, should prompt clinicians to evaluate for cardiac sarcoidosis using continuous ambulatory ECG (Holter) monitoring and echocardiography (7, 9).

Less common but clinically significant manifestations include:

- Pericarditis (acute or chronic)
- Endocarditis with valvular involvement
- Pulmonary embolism (secondary to venous stasis)
- Infiltrative cardiomyopathy mimicking amyloidosis or Fabry disease

### 2.3 Diagnostic capabilities

Cardiac magnetic resonance imaging (CMR) is one of the most important tools for diagnosing cardiac sarcoidosis, primarily through the detection of late gadolinium enhancement (LGE) in the myocardium, which typically presents with a patchy and multifocal uptake pattern while preserving the endocardial border. Several studies have demonstrated an association between LGE and poor prognosis (12).

Positron emission tomography with 18F-fluorodeoxyglucose (18F-FDG PET) is most useful for visualizing areas of myocardial inflammation and monitoring response to immunosuppressive therapy. The presence of 18F-FDG uptake and perfusion defects on PET has been associated with a higher risk of cardiac death or ventricular tachycardia. In particular, patients with focal inflammation of the right ventricle had a fivefold higher event rate compared to those with normal perfusion and metabolism, suggesting that focal right ventricular involvement may serve as a marker of more severe disease (5, 13).

Advanced cardiac imaging includes cardiovascular magnetic resonance and 18F-fluorodeoxyglucose positron emission tomography. Indications for advanced imaging include the following (14, 15, 17):

Patients with confirmed extracardiac sarcoidosis and one or more of the following criteria:

- One or more of the following symptoms: significant palpitations lasting more than 1–2 weeks, a history of presyncope or syncope
- One or more of the following ECG abnormalities: complete left or right bundle branch block, unexplained pathological Q waves in two or more leads, persistent first-, second-, or third-degree atrioventricular block, or sustained/non-sustained ventricular tachycardia
- One or more of the following echocardiographic abnormalities: regional wall motion abnormalities, ventricular aneurysm, basal septal thinning, or left ventricular ejection fraction below 50%

Patients without extracardiac sarcoidosis presenting with one or more of the following criteria:

- Unexplained Mobitz type II second- or third-degree atrioventricular block in adults under 60 years of age
- Sustained monomorphic ventricular tachycardia in the absence of any known etiology

Patients with extracardiac sarcoidosis who lack current symptoms or signs of cardiac involvement should be followed prospectively with serial (e.g., annual) clinical assessments and ECG monitoring for early detection of possible cardiac manifestations.

The main challenge in diagnosis lies in the low sensitivity of endomyocardial biopsy, which results from the patchy distribution of granulomatous inflammation. Pathognomonic granulomas containing multinucleated giant cells can be detected in only about 20% of biopsy samples, owing to their uneven distribution, which may involve any myocardial layer. The regions most frequently affected are the free wall of the left ventricle, followed by the interventricular septum, right ventricle, and occasionally the atrial wall (16, 18).

At the same time, imaging methods lack sufficient specificity for definitive diagnosis. Laboratory findings are also non-pathognomonic and may include anemia, leukopenia, or elevated erythrocyte sedimentation rate (19).

Transthoracic echocardiography is the least sensitive diagnostic tool for cardiac sarcoidosis, though it is specific, and observed abnormalities can assist in identifying cardiac dysfunction in patients with extracardiac sarcoid granulomas. Moreover, since left ventricular ejection fraction is one of the most important prognostic indicators, this relatively inexpensive imaging technique plays a role in patient management after diagnosis (20). Prospective studies indicate that newer indices, such as global longitudinal strain, may provide superior diagnostic and prognostic information compared with traditional transthoracic echocardiography (21).

A diagnosis of cardiac sarcoidosis may be classified as definite or indeterminate.

A definite diagnosis is established when non-caseating granulomas are identified in myocardial tissue without evidence of alternative causes. As histological findings are not pathognomonic, some experts consider the diagnosis “highly probable” in the presence of non-caseating granulomas.

Indeterminate cardiac sarcoidosis includes categories of high probability, probable, and possible. This term may be used when diagnostic uncertainty remains (16). Patients with clinically manifest cardiac sarcoidosis carry approximately a 10% risk of sudden cardiac death within 5 years of follow-up, whereas the risk in subclinical cases remains unknown. One of the direct complications of cardiac sarcoidosis is ventricular arrhythmia, which is a strong predictor of mortality (22, 23).

Major Diagnostic Criteria

- High-grade atrioventricular block or fatal ventricular arrhythmia
- Basal thinning of the interventricular septum or abnormal ventricular wall anatomy
- Abnormally high uptake on 67Ga citrate or 18F-FDG PET
- Left ventricular ejection fraction < 50%
- Improvement on gadolinium-enhanced MRI during follow-up

Minor Diagnostic Criteria

- Ventricular arrhythmias detected on ECG, abnormal Q waves, or deviation of the cardiac electrical axis
- Perfusion defects on myocardial perfusion scintigraphy
- Monocytic infiltration and moderate to severe interstitial myocardial fibrosis on endomyocardial biopsy

## 3 Pathogenesis hypotheses of cardiac sarcoidosis

Cardiac sarcoidosis (CS) is considered part of systemic sarcoidosis–a multisystem granulomatous disease of unclear etiology. Although the pathogenesis of CS has not been fully elucidated, several key hypotheses have been proposed, based on immunological, genetic, and environmental mechanisms (24, 25).

The prevailing concept suggests that in genetically predisposed individuals, exposure to an unidentified exogenous antigen triggers an abnormal immune response, leading to activation of CD4^+^ T lymphocytes and macrophages. Due to the perpetual nature of active inflammation in many cases of chronic sarcoidosis, perhaps a more appropriate term may be autoinflammatory disorder or that sarcoidosis involves an auto inflammatory response to stimulatory agents. (19). Granulomatous inflammation is primarily mediated by Th1-associated cytokines (IFN-γ, IL-2), although some studies have also reported a role for the Th17 response (1).

Potential triggers include mycobacterial and propionibacterial antigens. DNA from *Mycobacterium tuberculosis* and *Propionibacterium acnes* has been detected in sarcoid granulomas, including cardiac tissue, although a causal relationship has not yet been confirmed (25).

Genetic susceptibility to sarcoidosis, including its cardiac form, has been associated with certain HLA alleles such as HLA-DRB1 and DQB1, particularly in Japanese and Northern European populations (5). In recent years, polymorphisms in BTNL2, ANXA11, and TNFα genes have also been linked to an increased risk of sarcoidosis (6, 23, 26).

**FIGURE 2:**
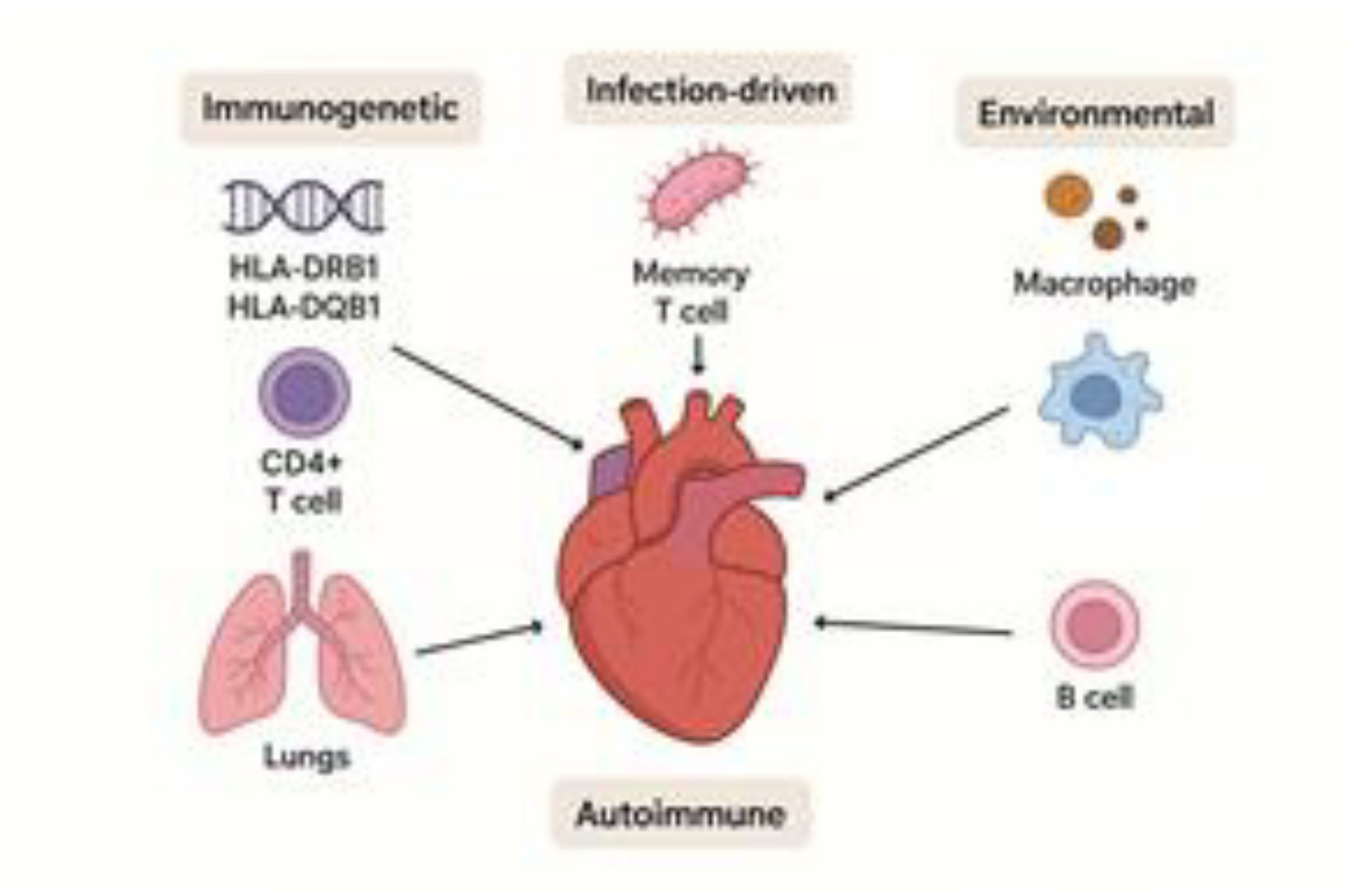
Hypotheses of cardiac sarcoidosis development.

Some researchers have proposed an autoimmune mechanism, in which autoantibodies directed against myocardial structural proteins, including β1-adrenoceptors and myosin, contribute to cardiac tissue injury (14, 15). This hypothesis is supported by the presence of inflammatory fibrosis and myocarditis in biopsy specimens even in the absence of systemic manifestations (Figure 2).

Myocardial fibrosis and remodeling may result either from active granulomatous inflammation or from isolated chronic injury. This duality explains the diagnostic challenge observed in the late stages of cardiac sarcoidosis (CS), when granulomas are no longer present and the clinical and imaging features resemble dilated or arrhythmogenic cardiomyopathy (23, 27, 28).

## 4 Immunopathological studies in cardiac sarcoidosis: cellular mechanisms, markers, and therapeutic targets

### 4.1 Key immune cells in the pathogenesis of pulmonary and cardiac sarcoidosis

Current evidence suggests that Th17.1 cells play a leading role in the pathogenesis of pulmonary sarcoidosis (29). Their levels are elevated in inflammatory foci within pulmonary tissue, where Th17.1 cells are found both at the periphery and in the center of granulomas (30, 31). Moreover, high frequencies of CCR4^–^CXCR3^+^ Th17.1 memory cells have been observed in lung-draining mediastinal lymph nodes and bronchoalveolar lavage (BAL) fluid (32).

Analysis of cardiac tissue from patients with cardiac sarcoidosis has revealed increased expression of cells co-expressing transcription factors TBX21 and RUNX1 (or TBX21 and AOAH)–a signature characteristic of Th17.1 cells–compared with control samples (33). Thus, in both pulmonary and cardiac sarcoidosis, Th17.1 cells migrate to sites of inflammation, promoting granuloma formation and the activation of tissue macrophages and fibroblasts–key effector cells likely contributing to adverse disease progression. These findings also highlight Th17.1 cells as a potential therapeutic target.

Studies of pulmonary sarcoidosis using both experimental models (34) and patient tissue samples (35) demonstrate high expression of Th2-associated cytokines–notably IL-4 and IL-13–which promote activation and differentiation of tissue macrophages toward the M2 phenotype. This may underlie chronic inflammation, granuloma formation, and tissue fibrosis. M2 macrophages are induced by Th2 cytokines (IL-4, IL-13) (36) and are typically associated with anti-inflammatory and reparative functions. However, in certain pathologies such as pulmonary sarcoidosis, these cells contribute to fibrotic tissue remodeling (37).

It has been proposed that the principal mechanism of fibrosis involves M2 macrophage–mediated fibroblast activation via a TGF-β-dependent pathway (38). The predominance of M2 macrophages in sarcoid granulomas has been confirmed in several recent studies. Shamaei et al. reported an increased number of M2 macrophages in pulmonary granulomas of sarcoidosis patients (35). An important role for M2 polarized alveolar macrophages in the pathogenesis of pulmonary fibrosis was also noticed by Pechkovsky et al., and IL-4 and IL-10 played the central part in shift to M2 phenotype of activated alveolar macrophages (39). In skeletal muscle affected by neuromuscular sarcoidosis, macrophages within granulomas exhibited increased expression of M2-specific markers (40). Furthermore, transcriptomic analysis of macrophages in cardiac sarcoidosis demonstrated upregulation of genes related to M2 polarization and glycolytic activation, whereas signaling pathways involved in phagocytosis, efferocytosis, autophagy, and cytokine regulation were downregulated compared with healthy controls (33).

Thus, M2 macrophages contribute to granuloma formation at various sites and promote fibrosis and chronic inflammation. Certain macrophage activation markers–lysozyme, neopterin, YKL-40 (human cartilage glycoprotein-39), soluble CD163 (sCD163), and serum amyloid A–may be elevated in patients with active sarcoidosis and reflect disease activity rather than diagnostic specificity due to their limited selectivity (41).

### 4.2 Cytokines and chemokines as markers of sarcoidosis

Studies of serum concentrations of CXCR3 ligands–CXCL9, CXCL10, and CXCL11–in various infectious and autoimmune diseases, including sarcoidosis, are valuable not only for diagnostic purposes but also for understanding pathogenesis and identifying new therapeutic approaches (42). In pulmonary sarcoidosis, granulomas show local accumulation of CXCL10, indicating its important role in recruiting effector cells to inflamed tissue and maintaining inflammation (43).

High serum levels of CXCL10 have been associated with reduced forced vital capacity (FVC), total lung capacity (TLC), and diffusing capacity for carbon monoxide (DLCO), whereas CXCL9 positively correlates with organ involvement (44). In contrast, CXCL11 levels were inversely correlated with FVC%predicted and FEV1%predicted, and directly correlated with the number of organs involved; high CXCL11 concentrations were also associated with increased patient-reported dyspnea scores (45).

In bronchoalveolar lavage fluid, elevated levels of all three CXCR3 ligands have been documented, with CXCL10 concentrations correlating with serum ACE levels, and CXCL11 levels closely associated with parenchymal lesions on high-resolution computed tomography (46).

Serum-soluble interleukin-2 receptor (sIL-2R, also known as sCD25) is another important biomarker of sarcoidosis. Its levels rise with T-cell activation in lymph nodes and inflammatory foci, as sIL-2R is shed from activated T cells into the circulation. This correlates with the accumulation of CD4^+^ T cells in BAL fluid and elevated serum sIL-2R concentrations in sarcoidosis patients (47).

Furthermore, regulatory T-cell (Treg) suppressive function inversely correlates with serum sCD25 levels, and elevated sCD25 has been reported in patients with thoracic lymphadenopathy (48). Receiver operating characteristic (ROC) analysis identified a serum sIL-2R threshold of 3550 pg/mL as optimal for diagnosing sarcoidosis (88% sensitivity, 85% specificity), outperforming serum ACE testing (62% and 88%, respectively) (49). A meta-analysis of ten studies (*n* = 1477) yielded pooled sensitivity and specificity values of 0.88 (95% CI: 0.75–0.95) and 0.87 (95% CI: 0.73–0.94), respectively (50).

High serum sIL-2R levels have also been proposed as a marker of chronic disease progression (51). Similar findings by Zhou et al. indicated that sIL-2R may serve as a predictor of spontaneous remission in patients with stage I pulmonary sarcoidosis (cut-off 1129.5 U/mL; sensitivity 50%, specificity 94.4%) and stage II disease (cut-off 1026.5 U/mL; sensitivity 66.7%, specificity 68.7%) (52). Elevated sIL-2R levels are also found in extrapulmonary sarcoidosis, including extranodal lymph node, splenic, and calcium metabolism abnormalities (53).

In cardiac sarcoidosis, patients with non-isolated disease demonstrate significantly higher sIL-2R levels than those with isolated cardiac involvement (54). Elevated serum sIL-2R correlates with an increased incidence of primary adverse outcomes and greater risk of subsequent cardiac events. However, this rise appears to reflect systemic inflammatory activity associated with lymph node inflammation rather than direct myocardial involvement (55).

CCL18 (macrophage inflammatory protein-4, MIP-4) is a potent chemoattractant for CCR8-expressing leukocytes and is secreted by alveolar and granuloma-associated macrophages in sarcoidosis (56, 57), leading to elevated circulating levels in affected patients. While CCL18 lacks diagnostic specificity, it serves as a useful marker for disease monitoring and prognosis, as its levels in BAL fluid negatively correlate with DLCO (58).

B-cell activating factor (BAFF), which regulates B-cell function, is increased in both serum and BAL fluid of sarcoidosis patients and correlates with ACE, lysozyme, and sIL-2R levels (59). Patients with three or more affected organs exhibit significantly higher serum BAFF levels than those with one or two involved organs. Elevated BAFF correlates inversely with vital capacity (%predicted) and DLCO (%predicted), and is associated with higher frequencies of skin and ocular involvement (60). Another study found elevated BAFF in sarcoidosis patients overall, but no significant differences between acute and chronic active forms (61). BAFF elevation, however, is not disease-specific, as it is observed in a range of autoimmune disorders (62).

Promising results have also been reported by Kiko et al., who demonstrated that serum B-type natriuretic peptide (BNP) measurement can distinguish patients with cardiac sarcoidosis from those with non-cardiac sarcoidosis (278.5 pg/mL vs. 21.8 pg/mL, respectively; BNP cut-off = 40 pg/mL; sensitivity 85.4%, specificity 68.1%) (54). In patients with cardiac sarcoidosis, BNP was a significant predictor of heart failure. Elevated baseline BNP may also be an independent predictor of future adverse events in patients without heart failure at diagnosis (63).

Finally, the oxidative DNA damage marker urinary 8-hydroxy-2′-deoxyguanosine (8-OHdG) has potential as a biomarker for inflammatory activity and therapeutic response in cardiac sarcoidosis. Elevated 8-OHdG levels in active disease correlate with steroid resistance and may predict cardiovascular mortality (64, 65).

Thus, multiple biomarkers are elevated in circulating blood and/or bronchoalveolar lavage fluid from patients with lung and cardiac sarcoidosis. The majority of the mentioned molecules may be related to specific pathogenetic mechanisms known to occur in sarcoidosis, but, unfortunately, they are also closely linked with different types of inflammatory reactions, that are typical for many other infection and autoimmune diseases. Future interventions should focus on clarifying which biomarker has potential as a reliable disease-specific indicator of sarcoidosis.

## 5 Therapeutic approaches in cardiac sarcoidosis

At present, cardiac sarcoidosis (CS) remains one of the most clinically significant manifestations of systemic sarcoidosis, characterized by a high degree of symptom heterogeneity and considerable diagnostic challenges. In this context, recent years have witnessed an intensification of both international and national studies aimed at improving diagnosis, risk stratification, and optimization of CS therapy (66, 67).

A stepwise treatment escalation algorithm is recommended, with systemic corticosteroids as first-line therapy. Corticosteroids remain the cornerstone of treatment for cardiac involvement, particularly in patients with evidence of active myocardial inflammation. Therapy is typically initiated with moderate-to-high doses, followed by gradual tapering based on clinical response, imaging findings, and arrhythmic burden.

Evidence-Based Pharmacological Therapies

A clear distinction between treatment modalities according to the strength of supporting evidence is essential.

Evidence-supported therapies.

Systemic corticosteroids represent first-line therapy and are supported by the largest body of clinical experience and observational evidence, demonstrating potential benefits in improving atrioventricular conduction abnormalities, suppressing inflammatory activity, and stabilizing or improving ventricular function when initiated early (68).

Steroid-sparing immunosuppressive agents, most commonly methotrexate and azathioprine, are widely used as adjunctive therapies to facilitate corticosteroid dose reduction and to maintain disease control. These agents are supported by observational studies and expert consensus and are considered standard components of long-term immunosuppressive regimens (67).

Second-line or adjunctive agents.

Mycophenolate mofetil is increasingly used as an alternative or adjunctive immunosuppressive agent, particularly in patients with intolerance or inadequate response to methotrexate or azathioprine. Although growing clinical experience supports its use, the evidence base remains predominantly observational, and its role is best considered as second-line therapy within a tailored immunosuppressive strategy (69).

Emerging biologic therapies.

Biologic agents, most notably tumor necrosis factor-α (TNF-α) inhibitors, are reserved for refractory cases with persistent inflammatory activity despite conventional immunosuppression. Their use is supported primarily by small observational cohorts and case series rather than randomized controlled trials. Consequently, these therapies should be explicitly recognized as emerging options and considered only in highly selected patients under specialist multidisciplinary supervision (54, 70).

Phenotype-Oriented Management: Inflammatory versus Fibrotic Disease

Active inflammatory disease.

In patients with imaging or clinical evidence of ongoing myocardial inflammation (e.g., FDG-PET positivity, recent conduction disturbances, declining ventricular function), immunosuppressive therapy plays a central role. Timely initiation and appropriate escalation of immunosuppression may lead to improvement or stabilization of atrioventricular conduction abnormalities, recovery of ventricular systolic function, and reduction in inflammatory burden. In this setting, anti-inflammatory treatment directly targets the underlying pathophysiology and may modify disease progression (70, 71).

In contrast, patients with predominantly fibrotic myocardial involvement, characterized by extensive late gadolinium enhancement on CMR with minimal or absent metabolic activity on FDG-PET, are less likely to derive functional benefit from intensified immunosuppression. Management in this phenotype shifts toward prevention of malignant ventricular arrhythmias, optimization of device therapy (including ICD and, where indicated, CRT), and comprehensive heart failure management in accordance with contemporary guidelines. In advanced cases, referral for advanced heart failure therapies may be warranted (71).

This phenotype-based approach underscores the importance of integrating imaging findings with clinical presentation to guide therapeutic decisions. Immunosuppressive escalation should be prioritized in inflammatory-active disease, whereas fibrotic-dominant disease necessitates a focus on arrhythmia prevention, device-based interventions, and long-term heart failure strategies. Such differentiation enables a more individualized, mechanism-driven treatment pathway and avoids unnecessary immunosuppression in patients unlikely to benefit.

In the field of therapy, the use of biological agents is currently under investigation. A phase II clinical trial of rilonacept is evaluating the efficacy of IL-1 blockade in combination with standard CS therapy. Systematic reviews confirm the role of corticosteroids as the cornerstone of treatment, whereas the effectiveness of additional use of standard immunosuppressants (such as methotrexate and azathioprine) still requires further validation.

Modern heart failure (HF) management strategies include, in addition to conventional therapy, biological agents (infliximab, adalimumab), standard HF medications, and mechanical circulatory support methods (LVAD implantation and heart transplantation) in terminal stages (67).

A phase II clinical trial investigating the use of rilonacept, a fusion protein that acts as a soluble decoy receptor for circulating IL-1α and IL-1β, has been initiated for the treatment of cardiac sarcoidosis. The aim is to assess its efficacy in combination with standard anti-inflammatory therapy, primarily glucocorticosteroids (ClinicalTrials.gov Identifier: NCT06660732). The results are expected upon completion of the clinical phase (24).

To date, glucocorticosteroids remain the mainstay of CS management. Their efficacy has been demonstrated in reducing inflammatory activity and preventing arrhythmias. The use of immunosuppressants (methotrexate, azathioprine) is considered an adjunctive option, though further validation through randomized controlled trials is required (5, 17).

Current therapeutic approaches to heart failure secondary to CS include standard pharmacological treatment (RAAS inhibitors, β-blockers, and diuretics), biological agents (infliximab, adalimumab), as well as mechanical circulatory support (LVAD implantation and heart transplantation) for patients with end-stage disease (72).

### 5.1 The role of the inflammasome and emerging therapeutic targets

Recent research has proposed the involvement of the inflammasome in initiating inflammatory reactions and maintaining granuloma formation in sarcoidosis (see Table 3).

**TABLE 3:** Potential therapeutic targets in cardiac sarcoidosis.

Target	Immune cells/pathway	Potential drug	Stage of investigation
mTOR	Macrophages, granulomas	Sirolimus, Everolimus	Off-label/protocol-based
Tubulin (mediates the interaction between the NLRP3 sensor and the ASC adaptor protein of the inflammasome complex)	Innate immune cells, cardiomyocytes, cardiac fibroblasts, endothelial cells of coronary arteries	Colchicine	Alternative therapy/adjunct to standard treatment
NLRP3	Innate immune cells, cardiomyocytes, cardiac fibroblasts, endothelial cells of coronary arteries	Dapansutrile	Off-label/protocol-based
IL-1β	Constitutively expressed: innate immune cells;		
Inducible: cardiomyocytes, cardiac fibroblasts, endothelial cells of coronary arteries	Canakinumab	Off-label/protocol-based	
IL-1β and IL-1α	Constitutively expressed: innate immune cells;		
Inducible: cardiomyocytes, cardiac fibroblasts, endothelial cells of coronary arteries	Rilonacept	Phase II (rilonacept)	MAGiC-ART study
IL-1Ra	Monocytes, macrophages, epithelial cells, fibroblasts, keratinocytes, endothelial cells, Th17	Anakinra	
IL-18	Constitutively expressed: innate immune cells;		
Inducible: cardiomyocytes, cardiac fibroblasts, endothelial cells of coronary arteries	Tadekinig alfa	Off-label/protocol-based	
IL-6	Innate immune response, Th17	Tocilizumab	Case series
TNF-α	Monocytes, granulomas	Infliximab, Adalimumab	Off-label
JAK/STAT	CD4^+^ T cells	Ruxolitinib, Tofacitinib	Pre-clinical stage
CTLA-4	Treg dysfunction	Abatacept (experimental)	Individual case reports

Huppertz et al. demonstrated increased expression of activated NLRP3 inflammasome components, cleaved caspase-1, and IL-1β in bronchoalveolar lavage (BAL) cells and granulomas from patients with sarcoidosis (69). It is noteworthy that inflammasome components can be synthesized in various cardiac cell types in response to inflammation or ischemia. Elevated expression of inflammasome-related molecules has been observed in cardiomyocytes, cardiac fibroblasts, and endothelial cells of coronary arteries in a range of cardiac pathologies (24). Thus, it is reasonable to suggest that the inflammasome plays an important role in the initiation and maintenance of inflammation in cardiac sarcoidosis.

A wide range of pharmacological agents targeting specific molecules involved in the activation of the inflammasome cascade or its downstream products have been identified. One example is DAPANSUTRILE, an NLRP3 inhibitor, which–although not yet approved by the FDA–is currently undergoing clinical trials in patients with gouty arthritis (73) and chronic heart failure (74). While its role in sarcoidosis has not yet been elucidated, DAPANSUTRILE represents a promising candidate for future clinical evaluation.

As previously noted, several monoclonal antibodies directly inhibit inflammasome-related cytokines such as IL-1β and IL-18. Tadekinig alfa, an IL-18 inhibitor, has not yet received FDA approval but has demonstrated high efficacy in difficult-to-treat adult-onset Still’s disease (75). Tadekinig alfa may therefore represent a potential therapeutic option for refractory forms of sarcoidosis of various localizations, though its efficacy in this condition remains to be fully investigated.

Numerous agents inhibiting IL-1β or its receptors are currently FDA-approved for use in other diseases. The main representatives include rilonacept, anakinra, and canakinumab. Of these, anakinra is presently being tested in cardiac sarcoidosis (76, 77). Rilonacept and canakinumab, in turn, are being actively investigated for their efficacy in chronic heart failure (CHF), ischemic heart disease (IHD), and pericarditis (78). Canakinumab is currently in a phase II placebo-controlled trial for refractory sarcoidosis (NCT02888080), while rilonacept is likewise in a phase II placebo-controlled trial for cardiac sarcoidosis (NCT06660732).

Non-specific inflammasome inhibitors, such as colchicine, may also be employed in sarcoidosis management. Evidence suggests that colchicine may be used as an adjunct or alternative therapy in cases of articular involvement in sarcoidosis (79, 80). Moreover, recent reports indicate beneficial outcomes when colchicine is added to standard therapy for cardiac sarcoidosis (81).

Thus, clinical evaluation of drugs targeting different stages of inflammasome activation represents a highly promising direction in the development of novel therapeutic strategies for cardiac sarcoidosis.

Device Therapy Indications, Prognostic Imaging Markers, and Follow-up Strategy

Clear indications for device therapy, based on international consensus and guideline documents, include the following (82).

- Implantable cardioverter–defibrillator (ICD) for secondary prevention. ICD implantation is indicated in patients with previously documented life-threatening ventricular arrhythmias (sustained ventricular tachycardia or ventricular fibrillation) or following aborted sudden cardiac death
- ICD for primary prevention. ICD implantation should be considered in patients with left ventricular systolic dysfunction (LVEF ≤ 35%) despite optimal medical therapy, particularly when additional high-risk features are present, such as extensive late gadolinium enhancement (LGE) on cardiac magnetic resonance (CMR) imaging or inducible sustained ventricular tachycardia in selected cases.
- Cardiac resynchronization therapy (CRT). Indications for CRT follow standard heart failure guidelines, including symptomatic heart failure, LVEF ≤ 35% despite guideline-directed medical therapy, and significant intraventricular conduction delay (typically a wide QRS complex), with device selection guided by contemporary ESC recommendations.
- Role of electrophysiological study (EPS). EPS may be useful for risk stratification in selected intermediate-risk patients, such as those with unexplained syncope, conduction abnormalities, or myocardial scarring on imaging without previously documented sustained ventricular arrhythmias. Inducible sustained ventricular tachycardia during EPS may support ICD implantation in borderline cases.

Prognostic Imaging Markers: Expanded Discussion

- LGE burden and distribution on CMR. The presence and extent of LGE are strongly associated with an increased risk of ventricular arrhythmias and all-cause mortality. A higher LGE burden, as well as specific patterns of distribution (including transmural, subepicardial, or multifocal involvement), confers a particularly unfavorable prognosis. Quantitative assessment of LGE has been shown to provide prognostic information beyond a binary assessment of its presence or absence.
- Right ventricular involvement. Right ventricular (RV) involvement, including RV dysfunction and/or RV LGE, is associated with a higher arrhythmic burden and adverse clinical outcomes. Comprehensive assessment of RV structure and function should therefore be an integral part of CMR evaluation, especially in patients with clinical or imaging evidence of right-sided disease (11).
- Complementary prognostic value of FDG-PET. Fluorodeoxyglucose positron emission tomography (FDG-PET) enables the identification of active myocardial inflammation and complements CMR by distinguishing active inflammatory lesions from chronic fibrotic scarring. The coexistence of metabolically active inflammation and myocardial scar is associated with an increased risk of ventricular arrhythmias and adverse outcomes. Quantitative PET parameters, such as standardized uptake values and the extent and localization of FDG uptake, may further refine risk stratification, although uniform prognostic thresholds have not yet been established (28).

Follow-up Strategy for High-Risk Patients

- General principles. Follow-up of high-risk patients should integrate regular clinical assessment, serial imaging, and systematic rhythm monitoring. The frequency and intensity of surveillance should be individualized according to baseline risk factors, including the extent of LGE, RV involvement, prior ventricular arrhythmias, and the degree of inflammatory activity on FDG-PET (70, 71).
- Serial FDG-PET imaging. In clinical practice, follow-up FDG-PET is often performed approximately 6 months after initiation or modification of immunosuppressive therapy to assess treatment response, with subsequent scans at 6–12-month intervals in clinically stable patients. Reduction or resolution of pathological FDG uptake is commonly used as a marker of inflammatory control and therapeutic efficacy (83).
- Serial CMR. CMR is recommended at baseline and should be repeated when there is a change in clinical status. While optimal intervals for routine follow-up are not firmly established, repeat CMR may be appropriate after several months in patients with disease progression, new arrhythmic events, or therapeutic escalation, to reassess ventricular function and myocardial scar burden.
- Rhythm monitoring. Patients at high arrhythmic risk should undergo prolonged rhythm surveillance, including continuous monitoring via implanted cardiac electronic devices when present. In patients without an ICD or pacemaker but with suspected intermittent arrhythmias, implantable loop recorders or extended external monitoring may be considered. Early detection of silent ventricular arrhythmias, progressive conduction disease, or atrial fibrillation may have important therapeutic implications (71).

Practical Considerations for Integrated Risk Stratification

An integrated approach combining left ventricular ejection fraction, LGE characteristics on CMR, inflammatory activity on FDG-PET, and relevant clinical features (such as prior ventricular arrhythmias, syncope, or atrioventricular conduction disturbances) provides superior risk stratification compared with any single parameter alone. LGE reflects the arrhythmogenic fibrotic substrate, FDG-PET identifies active inflammation, and assessment of right ventricular involvement further refines prognostic evaluation.

Electrophysiological study should be reserved for carefully selected cases in which the results are likely to influence management decisions, particularly regarding ICD implantation, and should ideally be discussed within a multidisciplinary team (70).

#### 5.1.1 Indications for referral to advanced heart failure therapies

Clear indications for referral to advanced heart failure therapies should be recognized early in the disease course. Referral to a specialized advanced heart failure and transplant center is warranted in patients with: Refractory heart failure, defined as persistent New York Heart Association (NYHA) class III–IV symptoms despite optimized guideline-directed medical therapy and appropriate immunosuppressive treatment.

Recurrent malignant ventricular arrhythmias, including recurrent sustained ventricular tachycardia or ventricular fibrillation despite optimal anti-arrhythmic therapy, catheter ablation where appropriate, and implantable cardioverter–defibrillator (ICD) protection.

Progressive disease despite optimal medical and immunosuppressive therapy, characterized by worsening ventricular dysfunction, increasing arrhythmic burden, or hemodynamic instability, even in the absence of active inflammation on imaging.

In such patients, timely evaluation for advanced heart failure therapies, including mechanical circulatory support and heart transplantation, is essential to avoid late referral and adverse outcomes.

Long-term systemic corticosteroid therapy has important implications for peri-operative risk in patients undergoing advanced heart failure interventions or heart transplantation. Chronic corticosteroid exposure is associated with an increased risk of peri-operative complications, including:

Infectious complications, due to sustained immunosuppression and impaired host defense mechanisms; Delayed wound healing and impaired tissue repair, related to steroid-induced alterations in collagen synthesis and inflammatory response; Metabolic and hemodynamic effects, including glucose dysregulation, hypertension, and adrenal suppression, which may complicate peri-operative management.

These factors necessitate careful pre-operative risk assessment, optimization of immunosuppressive regimens, and close multidisciplinary collaboration between heart failure specialists, transplant surgeons, and infectious disease teams. Where feasible, minimization of corticosteroid dose prior to surgery should be considered, balancing surgical risk against the risk of disease reactivation.

Cardiac magnetic resonance (CMR) and fluorodeoxyglucose positron emission tomography (FDG-PET) provide important complementary information in the post-transplant setting. CMR allows assessment of graft ventricular function and detection of myocardial scar or edema, while FDG-PET is particularly valuable for identifying metabolically active inflammation within the allograft. The combined use of these modalities improves sensitivity for detecting recurrent inflammatory activity compared with biopsy alone and may guide decisions regarding immunosuppressive adjustment (80, 84).

#### 5.1.2 Limitations of endomyocardial biopsy

Endomyocardial biopsy has limited sensitivity for detecting recurrent disease due to the patchy and focal nature of myocardial involvement. Sampling error remains a significant limitation, and a negative biopsy does not reliably exclude low-grade or regional inflammatory recurrence.

Several diagnostic criteria are widely accepted in clinical practice. The Japanese Ministry of Health, Labor and Welfare (MHLW) criteria, updated by the Japanese Circulation Society (JCS), use major and minor clinical findings and imaging features to establish cardiac sarcoidosis, allowing a clinical diagnosis even without histological confirmation in selected cases. The Heart Rhythm Society (HRS) consensus criteria similarly incorporate clinical and imaging data but generally require histological evidence of extracardiac sarcoidosis in the absence of positive myocardial biopsy. The World Association of Sarcoidosis and Other Granulomatous Disorders (WASOG) criteria provide a categorization of likelihood based on clinical, imaging and histological features. Variations among these frameworks reflect differences in emphasis on histology and diagnostic combinations and contribute to heterogeneity in reported cohorts and clinical practice (28).

### 5.2 Heart transplantation in cardiac sarcoidosis: outcomes and prognosis

Heart transplantation has remained the most effective treatment for patients with end-stage heart failure for several decades (85). Between 1992 and 2024, a total of 98,176 heart transplants were performed, and over the past decade, the annual number has increased from approximately 2,500 to 5,000 procedures (86).

An analysis of the United Network for Organ Sharing (UNOS) registry (1987–2019) identified 227 recipients with cardiac sarcoidosis among 63,947 heart transplant patients (84). The 39th Annual Adult Heart Transplant Report of the International Society for Heart and Lung Transplantation (ISHLT), covering data from January 1992 to June 2018, focused on restrictive cardiomyopathy (RCM) phenotypes as indications for transplantation. The frequency of cardiac sarcoidosis as a cause of cardiomyopathy among heart transplant recipients doubled between 1992–2009 and 2010–2018, rising from 3.3%–3.5% to 6.2%, respectively. It is important to emphasize that underdiagnosis of sarcoidosis likely persists, and both its prevalence and potential impact on post-transplant outcomes may be underestimated (87).

Indications for heart transplantation in patients with cardiac sarcoidosis do not differ from routine indications and include severe manifestations of heart disease despite therapy for heart failure, antiarrhythmic treatment, and sarcoidosis treatment (88):

- Class III–IV heart failure (NYHA) and VO2 peak < 12 ml/kg/min when beta-blocker therapy is included or VO2 peak < 14 ml/kg/min if no beta-blockers are used. The cardiomyopathy phenotype may vary: dilated with LV ejection fraction < 30%, or restrictive;
- Severe life-threatening ventricular arrhythmias (sustained ventricular tachycardia or recurrent ventricular tachycardias), frequent appropriate shocks from an implantable cardioverter-defibrillator (if present);
- Patient’s ability to follow medical recommendations and adhere to medication regimen;
- Absence of standard contraindications to heart transplantation or specific ones related to extracardiac manifestations of sarcoidosis (primarily lung involvement: pulmonary fibrosis with reduced lung function or development of high irreversible pulmonary hypertension with PVR > 5 Wood units).
- An important aspect in heart transplantation for a patient with sarcoidosis is current or past glucocorticoid therapy. Prolonged glucocorticoid therapy creates conditions for developing several complications in the early postoperative period (89):
- Infectious complications, including opportunistic infections;
- Increased risk of pathological fractures due to osteoporosis;
- Development of steroid myopathy, which complicates rehabilitation;
- Steroid-induced diabetes mellitus, arterial hypertension;
- Delayed healing of surgical wounds.

Therefore, pre-transplant evaluation should involve thorough examination of patients who have received steroid therapy to identify potential complications and understand future risks. A team-based approach in preparing such patients will help choose the right strategy for preoperative preparation and postoperative care (82).

**FIGURE 3:**
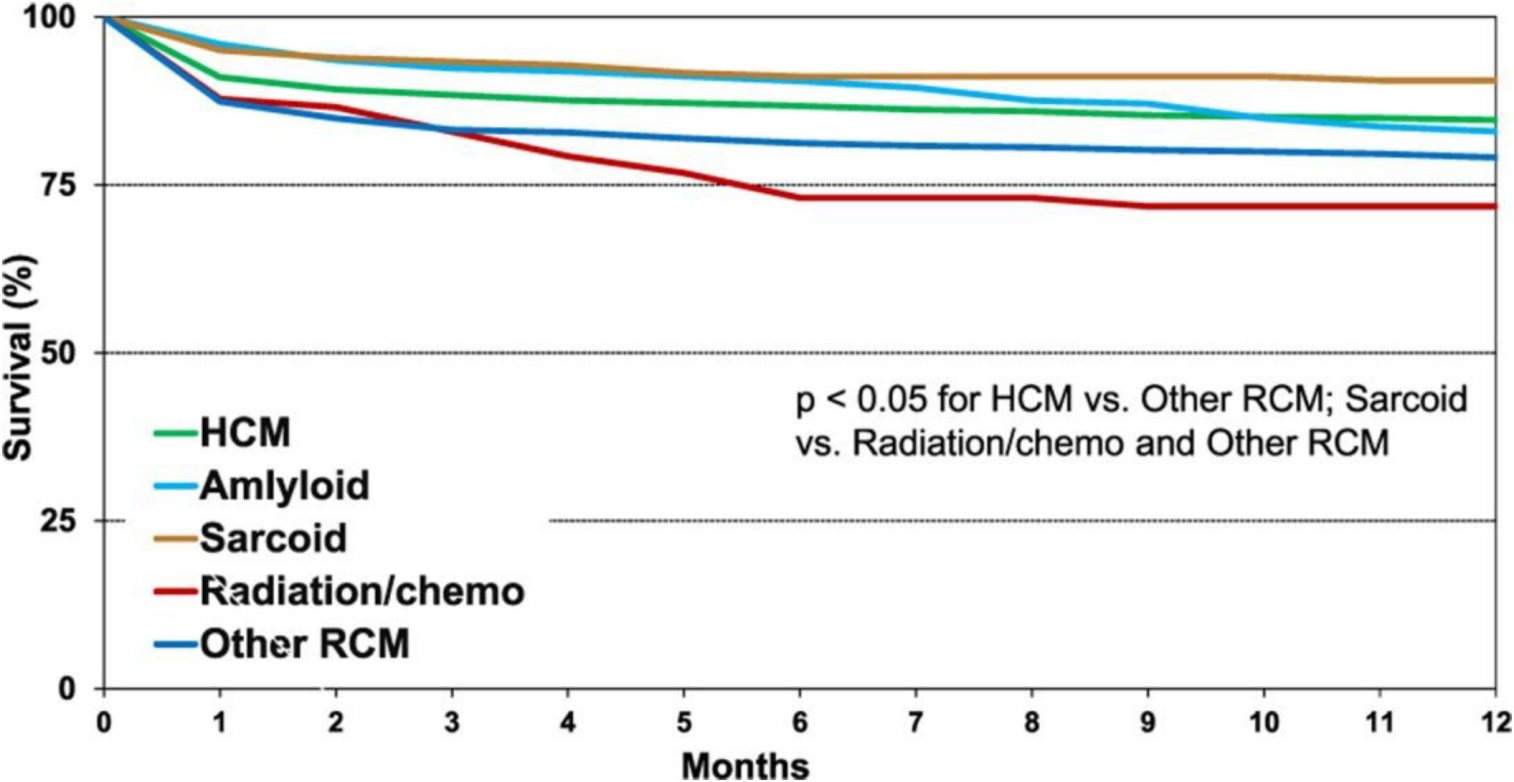
One-year Kaplan–Meier survival according to the underlying diagnosis leading to heart transplantation (transplants performed between January 1992 and June 2017): survival in patients with cardiac sarcoidosis – 90.4% (90).

The first post-transplant year remains associated with the highest mortality risk in all heart transplant recipients, primarily due to infectious complications, graft failure, rejection episodes, and multiple organ dysfunction. Among patients with restrictive cardiomyopathies, infection was the leading cause of death in recipients with cardiac sarcoidosis (43.8%). When comparing survival outcomes among heart transplant recipients with hypertrophic cardiomyopathy, amyloidosis, sarcoidosis, radiation-induced and/or anthracycline-related, and other restrictive cardiomyopathies, the highest 1-year and 5-year survival rates were observed in patients with cardiac sarcoidosis (see Figures 2, 3) (82).

Analysis of heart transplant recipient survival according to the UNOS registry from 1999 to 2020 (*n* = 41,447) demonstrated that patients with cardiac sarcoidosis and a restrictive cardiomyopathy (RCM) phenotype (*n* = 289) exhibited superior 5-year survival (87.7% [82.6%–91.4%] versus 77.2% [76.8%–77.7%], *p* = 0.004) and 10-year survival (73.4% [64.2%–80.6%] versus 59.5% [58.8%–60.1%], *p* = 0.002) compared with patients without RCM (82).

When comparing primary causes of death across groups, a higher proportion of patients with the restrictive phenotype of cardiac sarcoidosis died from fungal infections (primarily *Aspergillus*) or other respiratory system pathologies (pneumonia, chronic obstructive pulmonary disease, or restrictive lung disease) compared to those with other RCM variants or non-restrictive cardiomyopathies (4.3% versus 0.9%, *p* = 0.011). The increased rate of infectious complications as a cause of both mortality and hospitalization is likely associated with the frequent use of long-term glucocorticosteroid therapy prior to transplantation for the treatment of sarcoidosis.

In patients with cardiac sarcoidosis, the risk of allograft rejection after heart transplantation is comparable to that of recipients with other underlying diseases, particularly within the first postoperative year. Thereafter, the rejection risk remains similar or even decreases. According to the UNOS registry over a 20-year period (since 1999), the incidence of acute rejection episodes (odds ratio [OR] 0.95; 95% CI 0.66–1.35; *p* = 0.76) and hospitalizations due to rejection (OR 0.88; 95% CI 0.33–2.37; *p* = 0.81) was comparable between patients with cardiac sarcoidosis and other groups (86).

**FIGURE 4:**
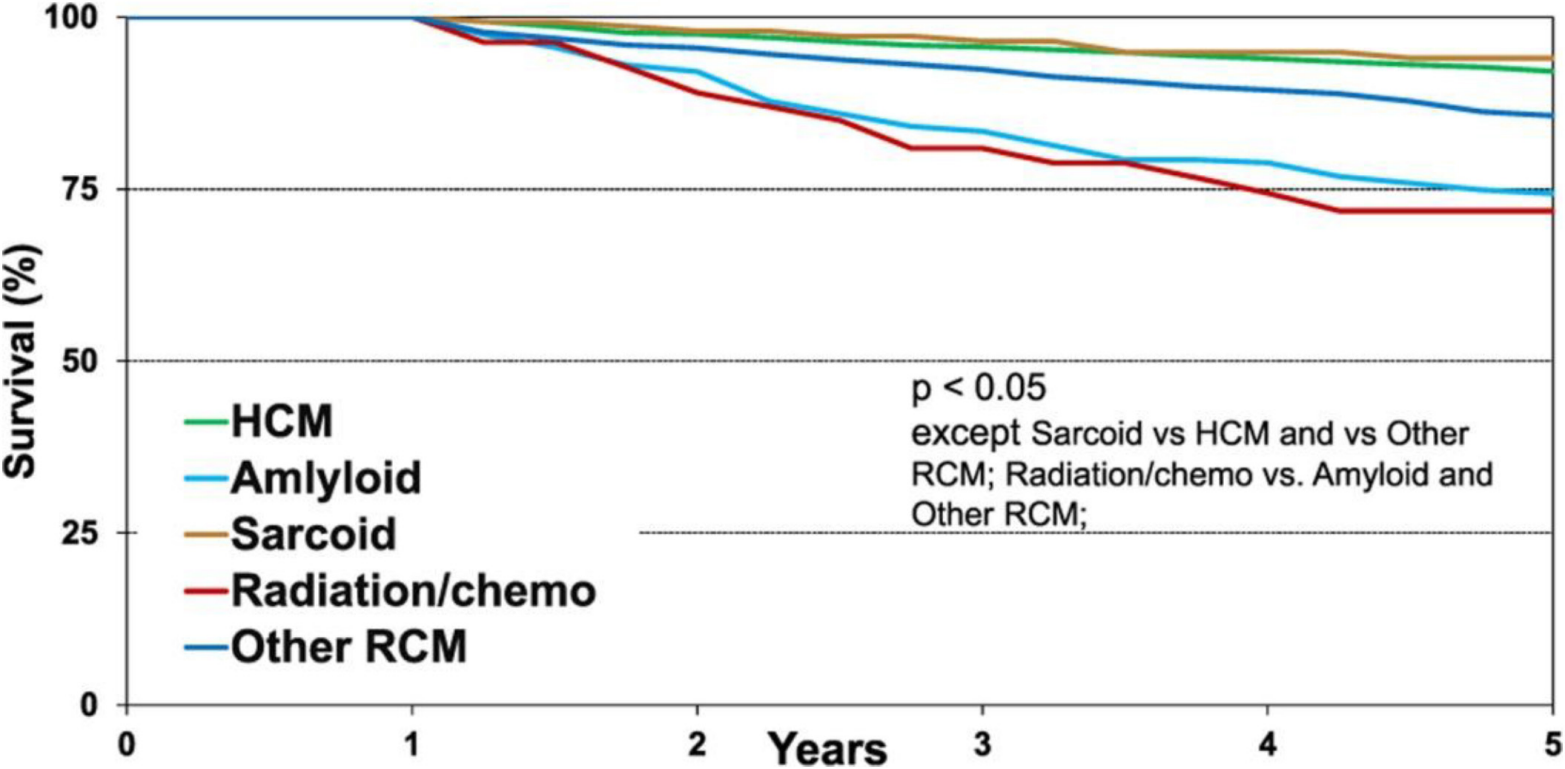
Five-year Kaplan–Meier survival conditional on 1-year post-transplant survival (transplants performed between January 1992 and June 2013): survival in patients with cardiac sarcoidosis – 94.0% (91).

Subsequently, a systematic review and meta-analysis revealed that within the first year following heart transplantation, the risk of acute cellular rejection was similar between patients with and without cardiac sarcoidosis. However, at 5 years (OR 0.38; 95% CI 0.03–0.72; I^2^ = 0%) and 10 years (OR 0.73; 95% CI 0.55–0.91), the risk of rejection was significantly lower in patients with cardiac sarcoidosis (Figure 4) (91).

Other post-transplant complications, such as allograft vasculopathy, malignancy, and infection-related hospitalizations, were comparable between patients with cardiac sarcoidosis and other recipient groups (86, 91).

In the post-transplant period, patients with cardiac sarcoidosis generally do not require major modifications of immunosuppressive therapy. However, according to the UNOS registry (1999–2020), the mean duration of corticosteroid therapy was 3.1 years, reflecting ongoing treatment of sarcoidosis after heart transplantation (92). A survey of 26 transplantation centers examining clinical practices in the management of cardiac sarcoidosis post-transplantation revealed that 62.5% of centers used prolonged steroid therapy, and 37.5% employed thymoglobulin induction (91).

The reported incidence of sarcoidosis recurrence in the transplanted heart varies across studies. According to the UNOS registry, no cases of recurrence were identified (89). A meta-analysis of nine cohort studies reported a recurrence rate of 5% among heart transplant recipients (28), whereas a survey of 26 transplantation centers found a mean recurrence rate of 37.5% (ranging from 0% to 50%) (28, 88). These discrepancies likely reflect differences in immunosuppressive strategies and, importantly, in the methods used for long-term monitoring of recurrence. Reliance on endomyocardial biopsy alone may be insufficient, and cardiac MRI and/or 18F-FDG PET/CT are recommended. In the study by Pandya et al., this combined approach was employed by 34.8% of transplant centers (81).

For diagnosing recurrence of sarcoidosis, cardiac MRI has advantages over 18F-FDG PET/CT because it is more readily available, does not require prior patient preparation, costs less, and offers high negative predictive value. If there are contraindications to performing MRI or ambiguous results occur (for example, in recipients with myocardial dysfunction due to acute or chronic graft rejection), then 18F-FDG PET/CT should be performed (22). The low rate of disease recurrence justifies recommending these investigations only on indication: appearance of rhythm disturbances, dysfunction of the transplanted organ, or progression of extrathoracic symptoms of sarcoidosis (91).

Post-transplant recurrence of inflammatory disease, although uncommon, has been reported and requires structured surveillance strategies. Early detection is clinically relevant, as recurrent inflammation may contribute to graft dysfunction, arrhythmias, or conduction abnormalities.

In summary, heart transplantation in patients with cardiac sarcoidosis is an effective therapeutic option that provides comparable outcomes over long-term follow-up and a more favorable prognosis. This applies to both the risk of rejection and the 5- and 10-year survival rates. Although the risk of sarcoidosis recurrence is generally low, it necessitates careful modification of immunosuppressive regimens and comprehensive monitoring of disease activity throughout the post-transplant period.

## 6 Conclusion

Cardiac sarcoidosis (CS) represents a clinically and pathobiologically important manifestation of systemic sarcoidosis, characterized by marked heterogeneity in presentation, a frequently occult course and substantial diagnostic challenges. Although clinically evident cardiac involvement is observed in only a minority of patients (≈5% clinically manifest), morphological cardiac involvement at autopsy is far more frequent (20%–30% in many series; reports from Japan indicate markedly higher rates), and the true burden of subclinical disease is almost certainly underestimated. Chronic systemic sarcoidosis develops in a sizeable minority of patients (approximately 10%–30%) and, when the heart is involved, is associated with disproportionate risks of morbidity and mortality.

Clinically, CS ranges from asymptomatic or mildly symptomatic disease to life-threatening presentations – sustained ventricular tachycardia, high-grade atrioventricular block, progressive heart failure and sudden cardiac death. The protean manifestations and patchy myocardial distribution of granulomas account for the low sensitivity of endomyocardial biopsy and for frequent diagnostic uncertainty. Consequently, a multimodal diagnostic strategy is essential: careful clinical assessment with targeted screening (ECG, ambulatory monitoring, transthoracic echocardiography) in all patients with extracardiac sarcoidosis; early recourse to advanced imaging (contrast-enhanced cardiac MRI with LGE and 18F-FDG PET) in the presence of symptoms, suggestive ECG/echo abnormalities, unexplained high-degree AV block in younger adults, or sustained ventricular arrhythmia. Imaging techniques have increased detection of subclinical CS substantially, but neither modality is fully specific – histology remains definitive when positive, and diagnostic algorithms must combine clinical, imaging and histological data.

Immunopathologically, CS appears to reflect a complex interplay of genetic susceptibility, antigenic triggers and dysregulated innate and adaptive immune responses. Accumulating data point to the involvement of Th1 and Th17-related pathways (notably Th17.1 cells), M2-polarized macrophages within granulomas, and chemokine/cytokine networks (CXCL9/10/11, sIL-2R, CCL18, BAFF and others) that correlate with organ involvement and disease activity. Emerging evidence implicates the inflammasome (NLRP3 → caspase-1 → IL-1β/IL-18) in granuloma maintenance and myocardial inflammation; inflammasome components are expressed by cardiomyocytes, fibroblasts and endothelial cells in inflamed myocardium. Several circulating and urinary biomarkers (sIL-2R, BNP, CXCL-family, CCL18, BAFF, urinary 8-OHdG) show promise for disease activity assessment and prognostication but currently lack sufficient specificity to replace clinical and imaging assessment.

Therapeutically, systemic glucocorticosteroids remain the mainstay of treatment for inflammatory activity in CS: they reduce inflammatory indices and are associated with decreased arrhythmic risk in many series. Conventional steroid-sparing immunosuppressants (methotrexate, azathioprine) are used as adjuncts, but high-quality randomized data validating their efficacy in CS are lacking. Biological therapies – notably TNF-α antagonists (infliximab, adalimumab) – have been employed as second-line options in refractory cases. The inflammasome/IL-1 axis has emerged as an appealing target: IL-1 blockers (anakinra, rilonacept, canakinumab) and IL-18 inhibitors (tadekinig alfa) are under active investigation, and NLRP3 inhibitors such as dapansutrile represent a mechanistically rational candidate for future trials. Non-specific agents with anti-inflammasome activity (colchicine) have also been reported to be of benefit in selected cases. For heart failure related to CS, guideline-directed heart-failure therapy remains fundamental; mechanical circulatory support (LVAD) and heart transplantation are appropriate for end-stage disease.

Heart transplantation for CS is associated with encouraging outcomes. Registry and cohort data demonstrate favorable short- and long-term survival (1-year survival ∼90% in some series; conditional 5-year survival ∼94%), and several analyses show comparable or even superior medium- and long-term survival versus other restrictive cardiomyopathies. The incidence of acute rejection does not appear to be increased long-term and may be lower at later time points. The reported recurrence rate of sarcoidosis in the graft varies widely between studies (meta-analytic pooled estimates ≈5% versus surveys reporting higher center-specific rates); such variability likely reflects heterogeneous immunosuppressive practices and differences in surveillance strategies (reliance on biopsy alone is insufficient – multimodal imaging surveillance with CMR and 18F-FDG PET is recommended). Infectious complications – particularly fungal and respiratory infections – represent a notable cause of morbidity and mortality in transplant recipients with sarcoidosis, plausibly linked to prolonged corticosteroid exposure pre- and post-transplant. An early clinical demonstration of a multimodal diagnostic strategy for cardiac sarcoidosis was provided by Mehta et al., who showed that a structured outpatient testing algorithm – combining symptom assessment, ECG, Holter monitoring, transthoracic echocardiography, and advanced imaging modalities such as MRI or PET – improved sensitivity for detecting cardiac involvement compared with conventional criteria alone. This study effectively presaged the contemporary multimodality diagnostic frameworks now incorporated into guideline recommendations (93).

In conclusion, cardiac sarcoidosis is a complex, multisystem disorder in which improved diagnostic sensitivity (largely driven by modern imaging), deeper immunopathological insight (notably regarding Th17.1 cells, M2 macrophages and the inflammasome) and the emergence of targeted anti-inflammatory therapies offer real potential to improve outcomes (94, 95). However, important gaps remain – in diagnostic standardization, biomarker validation and, critically, in high-quality randomized treatment data. Concerted multidisciplinary efforts combining robust mechanistic research, biomarker development and carefully designed clinical trials are required to translate recent scientific advances into better prognostication and evidence-based therapies for patients with cardiac sarcoidosis.
